# Mechanical Properties Variation in Wood—Plastic Composites with a Mixed Wood Fiber Size

**DOI:** 10.3390/ma16175801

**Published:** 2023-08-24

**Authors:** Hailong Xu, Yang Yang, Lifen Li, Baoyu Liu, Xiubo Fu, Xiaohui Yang, Yan Cao

**Affiliations:** 1 School of Data Science and Information Engineering, Guizhou Minzu University, Guiyang 550025, China; xu00hailong@163.com; 2 School of Mechanical Engineering, Chengdu University, Chengdu 610106, China; yangyang823@cdu.edu.cn; 3 College of Forestry, Guizhou University, Guiyang 550025, China; lifenli2011@163.com; 4 School of Marxism, Guizhou Minzu University, Guiyang 550025, China; liubaoyu1020@163.com; 5 School of Chemical Engineering, Guizhou Minzu University, Guiyang 550025, China; 18992714431@163.com; 6 School of Materials Science and Engineering, Guizhou Minzu University, Guiyang 550025, China; xiaohuiyang90@163.com

**Keywords:** wood fiber, size, mechanical properties, ROM, IROM

## Abstract

In this study, the influence of fiber particle size on the mechanical properties of a wood-–plastic composite (WPC) was investigated using a combination of experimental measurements and numerical modeling. Four different sizes of wood fibers (10–20 mesh, 20–40 mesh, 40–80 mesh, and 80–120 mesh) were used to reinforce high-density polyethylene (HDPE), either separately or in combination. The different sizes of fibers produced varying properties in the resulting composites. The smallest fiber size (80–120 mesh) resulted in the lowest flexural and tensile properties, but the highest impact strength (15.79 kJ/m^2^) compared to the other three sizes (12.18–14.29 kJ/m^2^). Using a blend of fiber sizes resulted in improved mechanical properties. Composites containing a mix of 20–40 mesh and 40–80 mesh fibers exhibited the best flexural (strength 74.16 MPa, modulus 5.35 GPa) and tensile performance (strength 48.27 MPa, modulus 4.30 GPa), while composites containing a mix of all four fiber sizes had the highest impact-resistant strength (16.08 kJ/m^2^). Several models, including the Rule of Mixtures (ROM), the Inverse Rule of Mixtures (IROM), and the Hirsch models, were used to predict the performance of WPCs. The ROM model was found to be the most accurate in describing the mechanical properties of WPCs reinforced with multi-size wood fibers, based on the sum squared error (SSE) analysis.

## 1. Introduction

Currently, the world faces a serious issue of resource depletion and environmental damage. This dilemma affects the global material system based on fossil fuels. To achieve sustainable development, it is better to develop natural and renewable resources to replace fossil materials. Biomass materials have emerged as a solution for this challenge.

Compared to industrial materials, biomass has advantages such as a green cleaning material. It can be used in complex environments and is a dynamic reaction system that cannot be easily reproduced in the laboratory. Biomass materials should also have excellent mechanical properties, such as strength, elasticity, hardness, fatigue resistance, wear resistance, and so on, while being easy to process into required shapes and forms.

Wood–plastic composites (WPC) have been rapidly promoted and applied due to their outstanding comprehensive performance, significant economic benefits, and alignment with China’s resource conservation and economic and industrial policies.

WPCs are primarily made of thermoplastic and wood fiber or powder, which is mechanically crushed and ground from scraps of pine, poplar and other wood. Other additives such as compatibilizers, flame retardants, and lubricants are also added. Filling plastic with wood powder reduces the density of products and gives it more woody properties. Natural fiber is a renewable resource that degrades naturally and does not harm the environment. WPCs are an economical and environmentally friendly material that is a new type of sustainable green composite material with broad application prospects. They have been widely used in various fields of material for construction because of their low cost, low abrasiveness, good mechanical properties, natural appearance, water resistance, biodegradability and ease of recycling and treatment. They are used for doors, window casings, outdoor desk–chairs, landscape wooden handrails, yacht wharf, outdoor garden sketches [1,2], and more. After being used, WPC products can be crushed into pellets and reprepared into new WPC materials and products for application.

When it comes to using WPCs in structural building, various factors affect their physical and mechanical properties. These factors include the wood species [3,4], the mass ratio of the wood fibers to plastic [5], the type and amount of coupling agent [6,7], the content of lubricant [8], the uniformity and cross-section design of the composites [9], and the processing temperature and pressure [10]. Another critical factor is the size of the wood fibers, which significantly influences the mechanical performance of WPCs. Stark and Rowlands [11] found that the aspect ratio has the most significant effect on the strength and stiffness in WPCs, while wood particle size does not significantly affect specific gravity. Garkhail et al. [12] have illustrated that there no improvement in the stiffness, tensile strength, and impact strength of flax/polypropylene (PP) composites was observed when the fibers exceeded the length of 6 mm [12]. Leu et al. [13] demonstrated that using recycled wood flour shorter than 125 μm (120 mesh) improved the tensile and flexural strength of WPCs, while reducing swelling due to water adsorption. Thumm and Dickson [14] studied wood pulp/PP composites and showed that a significant drop in composite flexural strength occurred when the length of pulp fiber was reduced from 1.3 mm to 0.8 mm. However, only the longest fiber (3.0 mm) presented a substantial increase in the Izod notched impact strength. Wang et al. found that composites prepared with 10–20 mesh wood fiber exhibited the best flexural properties but worst impact strength, and flexural strength and modulus decreased by 18% and 34% after exposing to UV-accelerated aging 2000 h [15].

The microstructure of fiber-reinforced polymer composites is complex and variable; therefore, it is vital to use a simple yet accurate mathematical model to understand the associated phenomena. Some commonly used models for mechanical properties include the Rule of Mixtures (ROM), the Inverse Rule of Mixtures (IROM), and the Hirsch model. Additionally, researchers have used other models to describe the mathematical properties of composites based on various parameters. Some of these models include the Proportional Hazards model, Kelly–Tyson model, Halpin–Tsai model, Bowyer–Bader model, Mori–Tanaka model, Levin model, Composite Cylinder Assemblage model, Shear–Lag model, Weibull Distribution model, and Hashin–Rosen model [16,17,18,19,20,21,22,23,24,25].

Cao et al. [26] measured and fitted the flexural, tensile, and impact properties of high-density polyethylene (HDPE) composites reinforced with hybrid fiber of flax and wood using the ROM, IROM, and Hirsch models. The IROM model was found to more accurate in describing the mechanical properties of the composites compared to the other models. The ROM and IROM models are based on Voigt’s assumption and Reuss’s assumption, respectively. In the former, both the matrix and fiber produce the same strain, and in the latter, the applied transverse stress is equal in both the matrix and the fiber [27,28,29,30].

(1) $$\mathrm{ROM}\;\mathrm{model}:\;\left\{\begin{array}{c} \sigma_{c}=\alpha \sigma_{f}V_{f}+\sigma_{m}V_{m}\\ E_{c}=\alpha E_{f}V_{f}+E_{m}V_{m} \end{array}\right.$$

(2) $$\mathrm{IROM}\;\mathrm{model}:\;\left\{\begin{array}{c} \sigma_{c}=\frac{\sigma_{f}\sigma_{m}}{\sigma_{f}V_{m}+\sigma_{m}V_{f}}\\ E_{c}=\frac{E_{f}E_{m}}{E_{f}V_{m}+E_{m}V_{f}} \end{array}\right.$$

Furthermore, the ROM model and IROM model were combined to form the Hirsch model.

(3) $$\mathrm{Hirsch}\;\mathrm{model}:\;\left\{\begin{array}{c} \sigma_{c}=\alpha \left(\sigma_{f}V_{f}+\sigma_{m}V_{m}\right)+\left(1-\alpha \right)\frac{\sigma_{f}\sigma_{m}}{\sigma_{f}V_{m}+\sigma_{m}V_{f}}\\ E_{c}=\alpha \left(E_{f}V_{f}+E_{m}V_{m}\right)+\left(1-\alpha \right)\frac{E_{f}E_{m}}{E_{f}V_{m}+E_{m}V_{f}} \end{array}\right.$$

The formulas shown above use variables *V*, σ, and *E* to represent the volume fraction, strength, and elastic modulus, respectively. The subscript “*f*” denotes fiber, “*m*” represents the matrix, and “*c*” denotes the composite. Parameter α is a factor that determines the stress transfer between the matrix and the fibers, and it also describes the behavior of short-fiber composites [26].

However, there is limited literature on the effect of multi-size wood fibers on WPC properties, and models are not frequently used to describe mixed wood fiber-reinforced thermoplastic composites. In this study, we investigated the effects of fiber size on the flexural, tensile, impact properties, and dynamic mechanical properties of WPCs. We used the ROM, IROM, and Hirsch models to simulate the mechanical properties of WPC and compare them to actual measurements. Our objective was to optimize the wood fiber size for manufacturing WPC and modify a suitable model to predict the mechanical property of WPCs, with the purpose of guiding the production of energy-saving and environmentally friendly WPC products.

## 2. Materials and Methods

### 2.1. Materials

**Figure 1:**
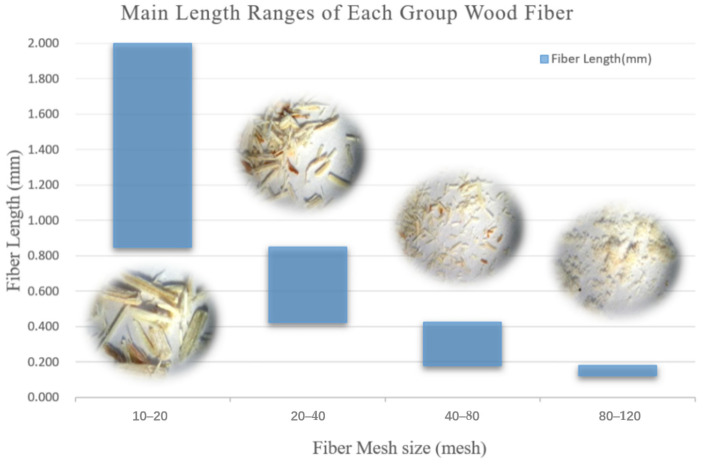
Fibers of different mesh sizes magnified 33 times.

HDPE particles (5000S) as the matrix in composites were purchased from Daqing Petrochemical Company in China. These particles have a melting flow index of 0.35 g/10 min and a solid density of 954 kg/m^3^. Poplar (*Populus alba*) wood fibers or particles were sieved into four groups: 10–20, 20–40, 40–80, and 80–120 mesh. The morphology of each group was magnified 33 times and shown in Figure 1. Maleated grafted polyethylene (MAPE) particles (CMG 9804) were used as a compatibility agent, and wax was used as a process lubricant. Both of these reagents were purchased from Tianjin Bochen Limited Company in China.

### 2.2. Preparation of Composites

Before mixing with the polymer, the wood fibers were dried in an oven set to 105 °C until the moisture content was less than 3.0%.

Dried HDPE, wood fibers, and other additives were mixed in a high-speed mixer for 15 min at 75 °C. The mixture was then compounded through a co-rotating twin-screw extruder (SJSH30, Nanjing Rubber-Plastic Machine Ltd., Nanjing, China), and then fed into SJ 45 mm conical counterrotating single-screw extruder to form samples of composite lumber. The screw speed was maintained at a constant 100 rpm, and the temperature was set between 150 °C and 175 °C during lumber extrusion. After cooling, the compounded mixture was broken into granules, which were then directly fed into a single-screw extruder (SJ45, Nanjing Rubber-Plastic Machine Ltd., Nanjing, China) to create the lumber. The cross-section of the lumber was 40 mm × 4 mm.

The composition mass ratio of WPC was shown in Table 1. In addition to single mesh (WPC 1, WPC 2, WPC 3 and WPC 4), we also prepared WF/HDPE composites (WPC 5, WPC 6, and WPC 7) with multi-size wood fibers.

### 2.3. Surface Observation of Different Wood–Plastic Composites Prepared

The surface morphology of different WPCs was observed using a stereomicroscope (XTL-350Z, Shanghai Changfang Optical Instrument Co., Ltd., Shanghai, China).

### 2.4. Mechanical Property Test

Samples were cut from WPC lumber and balanced for one week at ambient temperatures with a relative humidity of about 50%. The tests included flexural, tensile, impact, and dynamic mechanical properties.

(1) Flexural tests: Rectangular specimens with a length of 80 mm, width of 13 mm, and thickness of 4 mm were tested for flexural strength and modulus on an RGT-20A universal testing machine (Shenzhen, China). The test method and procedure were based on the standard ASTM D 790–03. The tensile testing speed was 5 mm/min, with a span of 64 mm. Each test was repeated six times.
(2) Tensile tests: Dumbbell-shaped specimens with a length of 165 mm, width of 20 mm, and thickness of 4 mm (measuring 12.7 mm in width in the narrowest portion) were tested for tensile strength and modulus on an RGT-20A universal testing machine (Shenzhen, China). The test method and procedure were based on the standard ASTM D-638. Six replicate tests were conducted.
(3) Unnotched Izod impact tests: Rectangular specimens with a length of 60 mm, width of 10 mm, and thickness of 4 mm were tested and performed at the impact speed of 2.9 m/s on samples with a span of 60 mm. The impact energy was 2 J. Ten replicate tests were performed.
(4) Statistical analysis: One-way analysis of variance (ANOVA) was performed to evaluate the statistical significance (*p* < 0.05) between the values of mechanical properties of WPCs with different sizes of wood fiber, and SPSS (Statistical Package for Social Sciences, version 19) software was used for statistics.
(5) Dynamic mechanical analysis: Rectangular specimens with a length of 40 mm, width of 10 mm, and thickness of 3 mm were tested on DMA-242 dynamic thermo-mechanical analyzer produced by NETZSH (Selb, Germany). Temperature spectrum scanning was carried out under the conditions of a three-point bending mode, frequency of 1 Hz, temperature ranges from −70 °C to 200 °C, and heating rate of 5 °C/min. Two replicate tests were conducted.

### 2.5. Mechanical Model Establishment and Application

The two elements (fiber and matrix) of the classical models (ROM, IROM, and Hirsch models) were extended to include two kinds of composites reinforced with different single-size wood fibers (for example, WPC 1 and WPC 4), which were prepared using the same matrix (the only difference between them was the size of the fiber). By using these two composites reinforced with different single-size fibers as the two elements, the mechanical properties of the composites reinforced with the corresponding multi-size fiber (WPC 5) were simulated.

Further, by using two composites reinforced with different multi-size fibers (WPC 5 and WPC 6) as the two elements, the mechanical properties of composites reinforced with the four corresponding multi-size fibers (WPC 7) were simulated.

To describe the mechanical properties of the composites, the ROM, IROM, and Hirsch models were applied, and their accuracy and reliability were compared using the sum squared error (SSE). The SSE, also called the “sum of squared error within-subject factors” and the “sum of squared residuals”, is the sum of the squared differences between estimated and experimental values. Take σ for example, the SSE was calculated as:

(4) $$\mathrm{SSE}=\sum_{i=1}^{i=7}{\left(\sigma_{model}^{i}-\sigma_{experiment}^{i}\right)}^{2}$$

where, *i* stands for the wood fiber type in the composites and reflects specific information about the observed values for each specimen.

(5) $$\mathrm{SSE}/\mathrm{AVE}=\sum_{i=1}^{i=7}{\left(\sigma_{model}^{i}-\sigma_{experiment}^{i}\right)}^{2}/(\sum_{i=1}^{i=7}{\sigma_{experiment}^{i}/7)}^{}$$

A lower value of SSE/AVE (the AVE is the average value of the experimental test data) indicates a better fit, meaning that the difference between estimated and experimental values is smaller, resulting in a more accurate model.

## 3. Results and Discussion

### 3.1. Surface Morphology of WPCs

**Figure 2:**
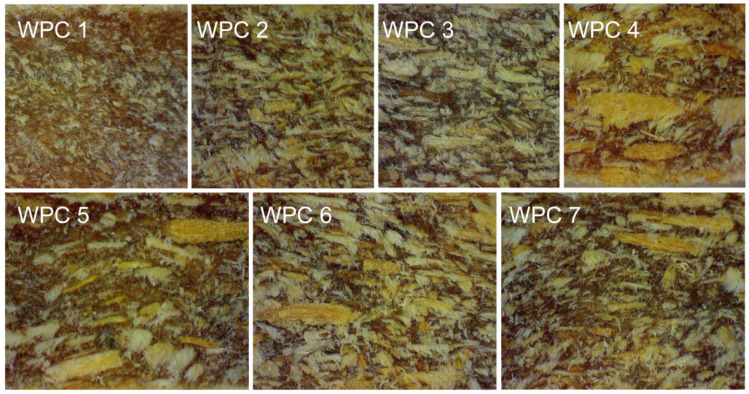
Surfaces of WPCs with different sizes of fiber using a stereomicroscope (48× enlargement) for WPC 1 (80–120 mesh), WPC 2 (40–80 mesh), WPC 3 (20–40 mesh), WPC 4 (10–20 mesh), WPC 5 (80–120 mesh plus 10–20 mesh,) WPC 6 (20–40 mesh plus 40–80 mesh), and WPC 7 (10–20, 20–40, 40–80, and 80–120 mesh).

The surface morphology of WPCs was observed using a stereomicroscope at a magnification of 48 times. Figure 2 displays the various group fibers in WPCs. The fibers in the extruded WPC were broken and altered in size, which could be attributed to fiber destruction during pretreatment, mixing with HDPE, and extrusion preparation under the shear and dispersion of the twin screws. Most fibers on the WPC surface were horizontally aligned and were parallel to the extrusion direction. However, in WPC 1, the fibers did not have good dispersion in the matrix, and their arrangement orientation was weak. This was mainly due to the small size of the 80–120 mesh wood fiber, which is powder-like in shape and prone to aggregate. A similar fiber distribution phenomenon can be observed in WPC 5. For composites reinforced with single-size fiber, the fiber distribution was relatively uniform in WPC 2 and WPC 3. On the other hand, for composites reinforced with multi-size fiber, wood fiber in WPC 6 had a fairly even distribution.

### 3.2. Mechanical Properties of WPCs

The flexural, tensile, and impact properties of composites are listed in Table 2.

The results showed that composites with single-size fibers had varying properties depending on the fiber size. The 80–120 mesh fibers presented the lowest flexural and tensile properties. Fibers that were too short (0.125–0.180 mm) had similar properties to powder and did not improve the flexural and tensile properties of WPCs due to their unfavorable aspect ratio. On the other hand, larger wood fibers a with better aspect ratio maintained more of the mechanical properties of wood and provided higher flexural and tensile properties. This is consistent with Bouafif et al. [31], who reported that the fiber origin significantly affects the properties of eastern white cedar, jack pine sawdust and black spruce sawdust reinforced HDPE composites, and higher fiber size produce higher strength and elasticity. However, if fibers were too large (10–20 mesh), they tended to entangle with each other and leave gaps without HDPE penetration. The best flexural and tensile properties were found in WPCs with 20–40 mesh fibers.

Compared to the tensile and flexural properties, the composites exhibited decreased impact strength as the fiber size increased, which agrees well with previous reports that large wood fiber size produces lower energy to break [31,32]. Composites reinforced with 80–120 mesh wood fibers showed the highest impact strength and demonstrated a significant difference compared to the other three groups. This may be due to the smooth surface and better flowability of small-size fibers which enable wood fibers to distribute more uniformly through the HDPE matrix and reduce the stress focus on the end of the fiber, thus retaining the toughness of the polymer matrix more effectively [33].

Composites containing multi-size fibers performed better than the average level of composites containing single-size fibers. For example, WPC 5 (the composite containing 80–120/10–20 mesh) presented higher flexural and tensile properties than the average value of WPC 1 (the composite containing 80–120 mesh) and WPC 4 (the composites containing 10–20 mesh). Similarly, the composite containing four groups of fibers (10–20, 20–40, 40–80, and 80–120 mesh) improved tensile strength and modulus by 10.2% and 31.3%, respectively, compared to the average value of four kinds of composite-containing fibers of 10–20 mesh, 20–40 mesh, 40–80 mesh, and 80–120 mesh.

In these seven groups, the composite that contained 20–40/40–80 mesh fibers showed the highest flexural and tensile performance. On the other hand, the composite that had four groups of fibers (10–120 mesh) presented the highest impact strength. This suggests that combining fibers of a suitable size is an effective method to enhance specific aspects of WPC performance. The improvement is attributed to the complementary benefits of both longer and shorter fibers. Smaller fibers uniformly distribute and fill the gaps between large fibers, while larger fibers provide stiffness to the composite.

In industrial production, sawing and sanding dust from the wood industry residue in tiny sizes are commonly used to produce WPCs, which helps reduce costs. However, this study indicates that incorporating some larger fibers can significantly improve the mechanical performance of WPCs.

### 3.3. Dynamic Mechanical Properties of WPCs

**Figure 3:**
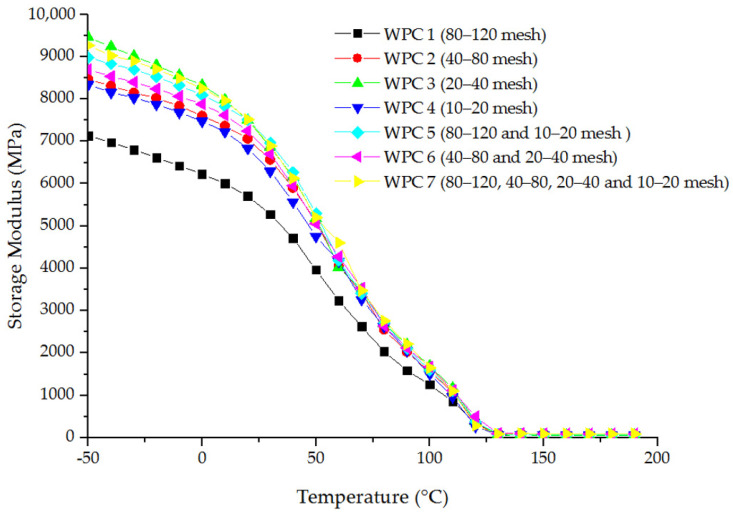
Relationship between the storage modulus and temperature of WPCs with different-size wood fibers.

The relationship between the storage modulus and temperature of polymer composites reinforced with fibers of different sizes were analyzed (Figure 3). The storage modulus of WPCs decreased with an increase in temperature across the entire temperature range. The difference in storage modulus between the composites was the most significant at −50 °C. Moreover, the storage modulus of a composite with 20–40 mesh wood fiber was the highest, while that of the composite with 80–120 mesh wood fiber was the lowest. The difference between these two composites was 2320.34 MPa. As the temperature increased from −50 °C to 0 °C, the storage modulus of the composites decreased gradually. However, as the temperature continued to rise, the decreasing speed of the storage modulus increased. When the temperature reached 125 °C, the storage modulus difference between the composites became smaller, and the storage modulus difference between composites containing two groups of fibers (20–40 and 40–80 mesh) with the highest-storage modulus and composites reinforced with 10–20 mesh wood fiber with the smallest storage modulus was 226.53 MPa. The size of the fiber in composites had a greater impact on the stiffness of the composites at a low temperature than at a high temperature.

The storage modulus of four single-size fiber-reinforced HDPE composites was also evaluated. The results showed that within the whole temperature range, the storage modulus of the composite with 80–120 mesh wood fiber was the lowest, while that of the composite with 20–40 mesh wood fiber was the highest. This result was consistent with the test results of static flexural strength; due to 80–120 mesh wood fibers being almost powdered, the continuity was damaged, the strength and stiffness were weakened, and the strengthening was affected, resulting in a significant reduction in the flexural property of the composite. However, the storage modulus was almost the same for the other three multi-size fiber-reinforced HDPE composites.

**Figure 4:**
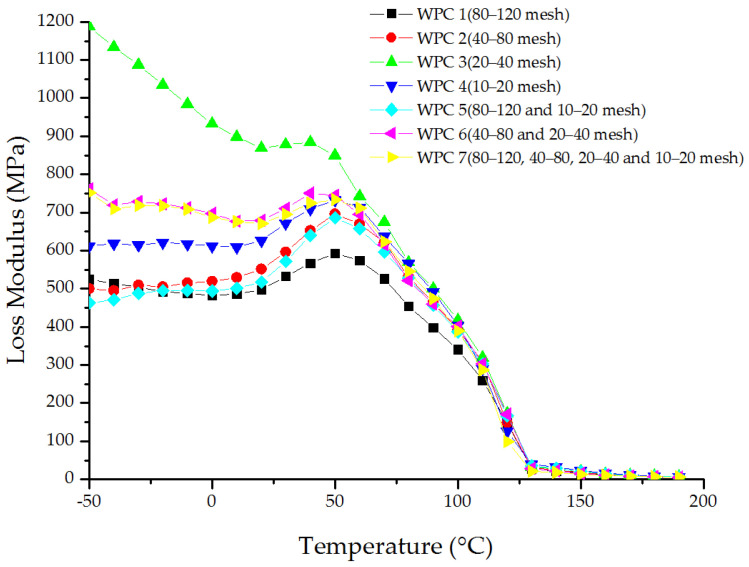
Relationship between the loss modulus and temperature of WPCs with different-size wood fibers.

The influence of the wood fiber size on the loss modulus of WPCs is demonstrated in Figure 4. As can be seen, the relaxation transition peaks appeared in succession between 25 °C and 55 °C. The HDPE composite reinforced with 20–40 mesh fibers had the lowest peak temperature of 29.6 °C, and the maximum loss modulus was 878.27 MPa. The HDPE composite reinforced with 20–40 and 40–80 mesh fibers had a peak temperature of 43.0 °C, and the corresponding loss modulus was also high, reaching 757.93 MPa. The 80–120 mesh fiber-reinforced HDPE composite had the lowest peak temperature of 51.3 °C, and the minimum loss modulus was 593.35 MPa. The peak temperature of the other composites was very similar, ranging from 49.7 °C to 50.9 °C, and the loss modulus ranged from 685.60 MPa to 733.42 MPa. As the wood fiber size increased, the required energy for the composites to begin melting first increased and then decreased, indicating that molecular movement became more difficult before becoming easier. This result supports the findings of the previous static impact test. The loss modulus of the composites decreased significantly when the temperature increased from 55 °C to 125 °C. Beyond 125 °C, the loss modulus of the composites became closer, ranging from 98.87 MPa to 172.33 MPa.

**Figure 5:**
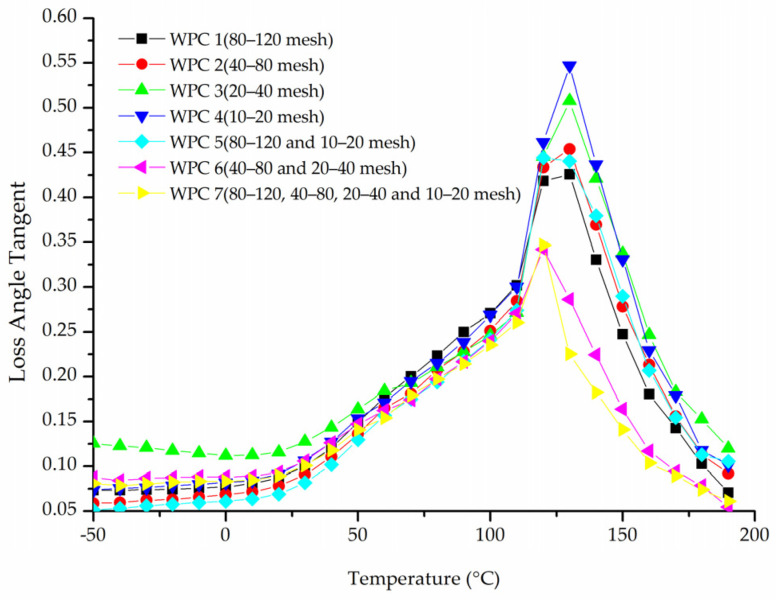
Relationship between the loss-angle tangent and temperature of WPCs with different-size wood fibers.

Figure 5 shows the influence of wood fiber size on the loss-angle tangent of WPCs. The tangent of the loss angle of composites gradually increased between −50 °C and 120 °C, indicating that the toughness of the composite improved with higher temperatures, and the improvement became more pronounced at higher temperatures. At around 125 °C, relaxation transition peaks appeared successively, and then the tangent of the loss-angle decreased sharply with increasing temperature.

### 3.4. Comparison between Modeled Values and Measured Values of Mechanical Properties

In order to compare the properties of WPCs, we used three different models (ROM, IROM, and Hirsch, see Equations (1)–(3)) to fit the properties of the composites with different multi-size fibers. It was assumed that WPCs contained multi-size wood fibers and were composed of two components: WPCs reinforced with one type of fiber and WPCs with another type of fiber, mixed at a volume ratio of 50/50. For example, the composite with 80–120 and 10–20 mesh sizes was composed of HDPE reinforced with 80–120 mesh size fibers and HDPE reinforced with 10–20 mesh sizes fibers, mixed at a volume ratio of 50/50. For the composite with four group of fibers (WPC 7, containing 10–20, 20–40, 40–80, and 80–120 mesh sizes), it was composed of the composite with 80–120 plus 10–20 mesh sizes (WPC 5) and the composite with 20–40 plus 40–80 mesh sizes (WPC 6), mixed at a volume ratio of 50/50. Based on this assumption, *V*_1_ = *V*_2_ = 0.5 in the ROM model and α is 1 when the two components of the composite were being mixed at a volume ratio of 50/50 [26]. While *V*_1_ = *V*_2_ = 0.25 in the ROM model and α is 1 when the four components of the composite were being mixed at a volume ratio of 25/25/25/25.

Equation (6) was built to calculate the strength and modulus of the composite composed of more than two reinforcement fibers.

(6) $$\left\{\begin{array}{c} \sigma_{No.m}=\sum_{i=1}^{n}\sigma_{No.i}V_{No.i}\\ E_{No.m}=\sum_{i=1}^{n}E_{No.i}V_{No.i} \end{array}\right.$$

where *V_No.i_* is the volume fraction of component *No.i* of the composite. The subscript “*m*” denotes the composite with mixed fibers.

Similarly, Equation (7) can be modified into IROM and Hrisch model styles. In which, *i* and *j* mean the component *No*.*i* of the composite and the component *No.j* of the composite, respectively.

(7) $$\left\{\begin{array}{c} \sigma_{No.m}=\frac{\prod_{i=1}^{n}\sigma_{No.i}}{\sum_{j=1}^{n}\frac{1}{n}\sigma_{No.j}}\\ E_{No.m}=\frac{\prod_{i=1}^{n}E_{No.i}}{\sum_{j=1}^{n}\frac{1}{n}E_{No.j}} \end{array}\right.$$

(8) $$\left\{\begin{array}{c} \sigma_{No.m}=\alpha \sum_{i=1}^{n}\sigma_{No.i}V_{No.i}+(1+\alpha )\frac{\prod_{i=1}^{n}\sigma_{No.i}}{\sum_{j=1}^{n}V_{No.j}\sigma_{No.j}}\\ E_{No.m}=\alpha \sum_{i=1}^{n}E_{No.i}V_{No.i}+(1+\alpha )\frac{\prod_{i=1}^{n}E_{No.i}}{\sum_{j=1}^{n}V_{No.j}E_{No.j}} \end{array}\right.$$

According to Equations (6)–(8), the performance of composites reinforced with multi-size wood fibers was calculated and listed in Table 3. While there were some deviations between the predicted and measured values, this is to be expected as composite properties cannot be accurately determined solely based on the volume or quantity ratio of the components. In this study, actual measured values were generally higher than predicted values, indicating a synergistic effect between different fiber sizes. For example, the use of both large and small fibers improved stiffness and tensile properties, respectively. For the composite with 10–20 and 80–120 mesh fibers (WPC 5), the average tensile modulus was 4.78 GPa, while the modeled tensile modulus of WPC 5 was 3.50–3.51 GPa, representing a 26.57–26.78% difference. Similarly, the average tensile strength of WPC 5 was 43.51 MPa, while the modeled tensile strength of WPC 5 was 39.56–39.66 MPa, representing an 8.85–9.08% difference.

**Figure 6:**
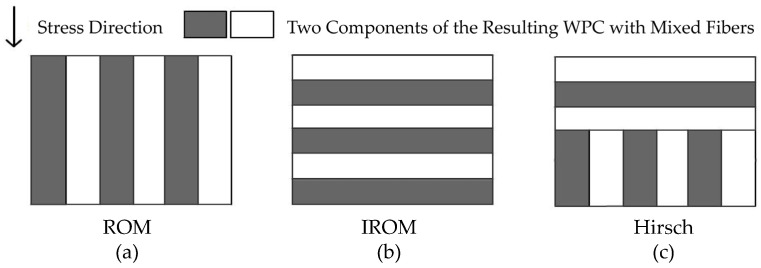
Schematic illustration of the ROM, IROM, and Hirsch models [34]. (**a**) The ROM model: isostrain and based on Voigt’s assumption; (**b**) TThe IROM model: isostress and based on Reuss’s assumption; (**c**) The Hirsch model: formed by combining ROM and IROM.

The SSE values for the three models were calculated using Equation (4) and the data from Table 3, which are presented in Table 4. The values of SSE/AVE for the three models were calculated using Equation (5) and the data from Table 2 and Table 3, which are presented in Table 5. It is noticeable that all three models exhibited similar SSE values and similar SSE/AVE values while describing the flexural, tensile, and impact performance of WPCs. The models showed the highest accuracy in simulating the flexural modulus and flexural strength, followed by impact strength, and the least accuracy in simulating tensile properties, especially tensile strength. The SSE value and the SEE/AVE value of the ROM model were smallest, indicating that it provides the most accurate description of the mechanical properties of WPCs. This result further confirms that Voigt’s assumption is better. As shown in Figure 6a, in Voigt’s assumption, both the matrix and fiber undergo the same strain, and it uses a sum of volume-weighted properties of the fibers and matrix to predict the properties of the composites [34].

The IROM model is based on Reuss’s assumption, in which the applied transverse stress is equal in both the fiber and the matrix, as shown in Figure 6b [34]. The Hirsch model is formed by combining ROM and IROM, as shown in Figure 6c [34]. However, in this research, fibers of different mesh sizes were randomly distributed through the WPC lumber, rather than being in one layer on top of another. Most fibers were horizontally oriented and parallel to the extrusion direction, as shown in Figure 2. The flexural and impact test applied stress perpendicular to the surface plane. While the ROM and Hirsch models considered the orientation of the fiber, IROM did not. Therefore, IROM was the poorest in fitting the property of WPCs. The accuracy of the simulation employing the Hirsch model was moderate, as it is formed by combining ROM and IROM.

**Figure 7:**
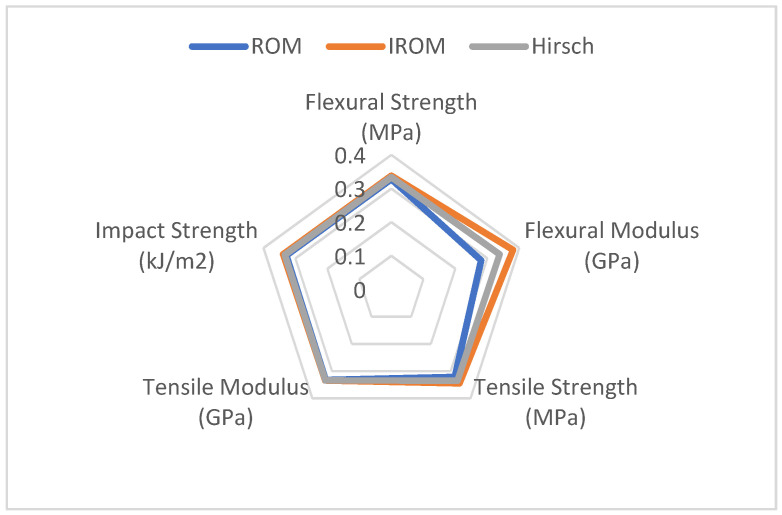
Radar chart of the accuracy of mechanical properties of WPCs simulated by ROM, IROM, and Hirsch.

Table 6 shows the normalized results of the SSE/AVE value for WPCs after being classified and normalized in mathematics. Figure 7 presents the radar chart of the accuracy of mechanical properties of WPCs simulated by ROM, IROM, and Hirsch. The smallest difference in the simulated results among the three models is for the tensile modulus, followed by flexural strength and impact strength. However, for the flexural modulus and tensile strength, the difference is relatively large, with the tensile strength showing the largest difference. The ROM model provides the best simulation for the flexural modulus and tensile strength, followed by Hirsch, while IROM is the least effective. The radar chart, which is closer to the center point, has the better fitting effect. Thus, the smaller the area surrounded by the model curve, the better the comprehensive effect in simulating the mechanical properties of WPCs. The area surrounded by the ROM model curve in Figure 7 is the smallest, indicating that it had the best comprehensive effect in simulating the mechanical properties of WPCs reinforced with multi-size wood fibers.

## 4. Conclusions

The size of wood particles affects the mechanical properties of wood–plastic composites. Small wood fibers (80–120 mesh) have lower flexural and tensile properties, but better impact strength compared to large wood fibers. Moderate-size (20–40 mesh) wood fibers contributed to the highest flexural and tensile properties. WPCs with multi-size wood fibers have better mechanical properties than those with single-size fibers. An ideal combination is mixed wood powder of 20–40 and 40–80 mesh to reinforce HDPE. To improve impact performance, fiber of sizes 10–20, 20–40, 40–80, and 80–120 mesh should be used uniformly, which can increase the average impact strength value of WPCs by 12.76%. The ROM model is more accurate than the IROM and Hirsch models in describing the flexural, tensile, and impact properties of WPCs reinforced with multi-size wood fibers.

Overall, this study analyzed the effects of mixed wood fiber size on the mechanical properties of WPCs, and found that the mechanical performance of WPCs prepared by certain-size fibers (0.180–0.425 mm) is better than that of WPCs prepared with longer or shorter fibers. Therefore, before the preparation of WPCs, appropriate process conditions need to be considered to produce wood fibers with of a suitable size. Alongside that, this study proposed models for predicting the mechanical properties. These models provide a theoretical basis for designing and preparing high-performance WPCs for applications in outdoor flooring, landscape architecture, exterior wall-hanging panels, decorative materials, and other fields.

## Figures and Tables

**Table 1:** Composition mass ratio of WPCs.

No.	HDPE	Additives	Wood Fiber			
			0.850–2.000 mm (10–20 Mesh)	0.425–0.850 mm (20–40 Mesh)	0.180–0.425 mm (40–80 Mesh)	0.125–0.180 mm (80–120 Mesh)
WPC 1	36	4	-	-	-	60
WPC 2	36	4	-	-	60	-
WPC 3	36	4	-	60	-	-
WPC 4	36	4	60	-	-	-
WPC 5	36	4	30	-	-	30
WPC 6	36	4	-	30	30	-
WPC 7	36	4	15	15	15	15

**Table 2:** Mechanical properties of WPCs.

Test	Wood Fiber Size (Mesh Size)	Flexure Test		Tensile Test		Impact Test
		Strength σ_F_ (MPa)	Modulus E_F_ (GPa)	Strength σ_T_ (MPa)	Modulus E_T_ (GPa)	Strength σ_I_ (kJ/m^2^)
1	80–120	67.39 ^d^ (0.14)	3.83 ^d^ (0.25)	37.69 ^d^ (0.63)	3.35 ^d^ (0.70)	15.78 ^ab^ (1.04)
2	40–80	70.44 ^c^ (0.95)	5.76 ^a^ (0.07)	40.49 ^c^ (1.07)	3.45 ^cd^ (0.35)	14.29 ^c^ (1.31)
3	20–40	73.43 ^a^ (1.00)	5.48 ^a^ (0.11)	46.48 ^a^ (0.54)	3.66 ^bcd^ (0.87)	14.79 ^bc^ (0.81)
4	10–20	71.14 ^bc^ (1.05)	5.12 ^b^ (0.09)	41.63 ^b^ (0.76)	3.46 ^cd^ (0.74)	12.18 ^d^ (0.87)
5	80–120 and 10–20	70.30 ^c^ (0.75)	4.85 ^c^ (0.06)	43.51 ^a^ (0.47)	4.78 ^a^ (0.41)	13.97 ^c^ (0.87)
6	40–80 and 20–40 (20–80)	74.16 ^a^ (0.89)	5.35 ^a^ (0.06)	48.27 ^a^ (0.77)	4.30 ^abc^ (1.18)	15.55 ^ab^ (0.73)
7	10–20, 20–40, 40–80, and 80–120 (10–120)	71.70 ^b^ (1.27)	5.08 ^b^ (0.09)	45.81 ^a^ (0.37)	4.57 ^ab^ (0.31)	16.08 ^a^ (1.11)

Mean values with the same letter for given property are not significantly different (*p* ≤ 0.05).

**Table 3:** Measured and modeled mechanical properties of WPCs.

Test	Mesh Size of the Wood Fiber			Flexure Test		Tensile Test		Impact Test
				Strength σ_F_ (MPa)	Modulus E_F_ (GPa)	Strength σ_T_ (MPa)	Modulus E_T_ (GPa)	Strength σ_I_ (kJ/m^2^)
5	80–120 and 10–20	Experimental Value		70.30	4.85	43.51	4.78	13.97
		Fitted Value	ROM	69.27	4.48	39.66	3.51	13.98
			IROM	69.21	4.38	39.56	3.50	13.75
			Hirsch	69.23	4.42	39.60	3.50	13.84
6	40–80 and 20–40 (20–80)	Experimental Value		74.16	5.35	48.27	4.30	15.55
		Fitted Value	ROM	71.94	5.62	43.49	3.46	14.54
			IROM	71.90	5.62	43.28	3.45	14.53
			Hirsch	71.92	5.62	43.36	3.45	14.54
7	10–20, 20–40, 40–80, and 80–120 (10–120)	Experimental Value		71.70	5.08	45.81	4.57	16.08
		Fitted Value	ROM	72.23	5.10	45.89	4.54	14.38
			IROM	72.18	5.09	45.77	4.53	14.37
			Hirsch	72.20	5.09	45.82	4.53	14.37

The value of α is 1 in the ROM model and 0.4 in the Hirsch model [26].

**Table 4:** SSE values of the three models for WPCs.

Mechanical Properties		ROM	IROM	Hirsch
Flexural Properties	Strength σ_F_ (MPa)	6.303	6.498	6.418
	Modulus E_F_ (GPa)	0.214	0.290	0.258
Tensile Properties	Strength σ_T_ (MPa)	37.725	40.500	39.374
	Modulus E_T_ (GPa)	2.341	2.359	2.352
Impact Strength	Strength σ_I_ (kJ/m^2^)	2.948	3.038	2.989

The value of α is 1 in the ROM model and 0.4 in the Hirsch model [26].

**Table 5:** SSE/AVE values of the three models for WPCs.

Mechanical Properties		ROM	IROM	Hirsch
Flexural Properties	Strength σ_F_ (MPa)	0.088497	0.091235	0.090112
	Modulus E_F_ (GPa)	0.042233	0.057231	0.050916
Tensile Properties	Strength σ_T_ (MPa)	0.869011	0.932934	0.906996
	Modulus E_T_ (GPa)	0.594378	0.598948	0.597171
Impact Strength	Strength σ_I_ (kJ/m^2^)	0.201052	0.207190	0.203848

The value of α is 1 in the ROM model and 0.4 in the Hirsch model [26].

**Table 6:** The normalized results of the SSE/AVE values for the three models.

Mechanical Properties		ROM	IROM	Hirsch
Flexural Properties	Strength σ_F_ (MPa)	0.327956	0.338103	0.333941
	Modulus E_F_ (GPa)	0.280842	0.380576	0.338582
Tensile Properties	Strength σ_T_ (MPa)	0.320794	0.344391	0.334816
	Modulus E_T_ (GPa)	0.331963	0.334515	0.333522
Impact Strength	Strength σ_I_ (kJ/m^2^)	0.328468	0.338496	0.333036

## Data Availability

Not applicable.
